# Retraction: The Prevalence of Ethical Conflicts and Influencing Factors Among Critical Care Nurses in China: A National Cross-sectional Study

**DOI:** 10.1097/jnr.0000000000000708

**Published:** 2025-10-28

The article entitled “The Prevalence of Ethical Conflicts and Influencing Factors Among Critical Care Nurses in China: A National Cross-sectional Study” published online in July 2025 in *The Journal of Nursing Research*, is being retracted due to ethical issues.
